# Techniques of Eyebrow Lifting: A Narrative Review

**DOI:** 10.18502/jovr.v15i2.6740

**Published:** 2020-04-06

**Authors:** Nasser Karimi, Mohsen Bahmani Kashkouli, Hamed Sianati, Behzad Khademi

**Affiliations:** ^1^ Eye and Skull Base Research Centers, The Five Senses Institute, Iran University of Medical Sciences, Tehran, Iran

**Keywords:** Blepharoplasty, Endoscopy, Esthetics, Eyebrow, Forehead, Lifting, Rejuvenation

## Abstract

None of brow lift techniques are completely satisfactory because of their limited effectiveness, lack of longevity, and potential complications. The aim of this study was to provide a comprehensive review of the literature on the pros and cons of the most popular techniques in brow and forehead lift. Relevant original articles in the PubMed database (English language) were sought using the search terms “eyebrow lift", “forehead lift", "periorbital rejuvenation", "eyebrow ptosis", "blepharoplasty and eyebrow change", "surgical eyebrow lift", and "non-surgical eyebrow lift", No date limitation was considered. Titles and abstracts were scanned to include the most pertinent articles. Subsequently, full texts of included articles (111 articles) were skimmed and finally 56 references were selected for the review. A narrative synthesis of data was finally undertaken with particular attention to the indications, techniques, and common complications of the eyebrow lift procedures. Ten popular techniques including two non-surgical methods (Botulinum toxin A and soft tissue fillers) were reviewed in this article. In general, non-surgical methods of forehead/brow lift are temporary, need less experience and correction would be easier should any complication occur. Surgical methods are divided into three categories: trans-blepharoplasty eyebrow lift, direct eyebrow lift, and trans-forehead eyebrow/forehead lift. Currently, the most popular method is the endoscopic forehead lift approach even though its longevity is limited. Direct brow-lift is particularly useful in patients with facial palsy and those who are more likely to be accepting of the scar (male gender, high forehead hair line).

##  INTRODUCTION

Although each operation is customized to the patient's individual senile changes, most patients looking for blepharoplasty procedure have a component of eyebrow ptosis with or without asymmetry.^[1]^ Therefore, the knowledge of surgical anatomy and interventions for brow and forehead rejuvenation are critical for an oculo-facial plastic surgeon.^[2]^ In contrast to upper and lower blepharoplasty, with a standard technique^[3,4]^ adopted and applied almost universally, a multitude of surgical techniques are available to address eyebrow and forehead. These may be combined with upper blepharoplasty for stable, long lasting, and natural results. Recognizing each patient's individual attributes and matching them to the ideal rejuvenation techniques will allow for maximum aesthetic benefit. For instance while an old man with few hair on head and multiple deep horizontal forehead wrinkles may be satisfactorily treated with mid-forehead brow lift with relatively lower cost and recovery period, a young woman with abundant hair benefits most from endoscopic lift. As such the aim of this review was to elaborate on the indications, contraindications, advantages, and disadvantages of the common and current techniques in the management of eyebrow/ forehead ptosis.

##  METHODS

A review of current available literature was performed using PubMed database. We limited our investigation to English-language journals. The keywords for initial data bank searches included "periorbital rejuvenation", "eyebrow ptosis", “eyebrow lift", “forehead lift", "blepharoplasty and eyebrow change", "surgical eyebrow lift", and "non-surgical eyebrow lift". There was no restriction on the date of publication. Titles were scanned by the senior author to include the most pertinent articles (233 articles). Abstracts were subsequently reviewed to select 111 articles to go through their full text. A narrative synthesis of data was finally undertaken, citing 54 articles and 2 books, with a special attention to the techniques of the eyebrow lift. We acknowledge the limitation that this is not a systematic review and we have certainly missed sound publications not indexed in Medline.

### Common and Current Methods of Brow Lift 

There are two categories of brow elevation and forehead lifts [Table 1]. Non-surgical methods of forehead/brow lift are temporary, require less experience, and in case of any complication, can be corrected easily. Surgical methods, on the other hand, include some with temporary effect (suture lift and trans-blepharoplasty approaches) and some with comparatively permanent effect (direct and trans-forehead approaches). Surgical techniques are generally more surgeon- and equipment-dependent and complications are more sophisticated.

**Table 1:** Advantages and disadvantages of the different techniques for the eyebrow lift


	Definition	**Indications**	**Contraindications**	**Advantages**	**Disadvantages**
Non-surgical methods					
Botulinum toxin A injection	To treat the depressor muscles of the brow with BTA	Desire to elevate the lateral eyebrow with a less invasive method	Hypersensitivity to BTA	Less invasive, less expensive, no major permanent side effect	Temporary effect of the central and lateral eyebrow, little effect on medial eyebrow
Soft tissue fillers	Injection of filler in the lateral eyebrow to promote support of the retro-orbicularis oculi fat	Improving the elevation of the eyebrow tail in cases where BTA provides insufficient eyebrow lifting	One eye patients	Fillers can enhance eyebrow contour and volume	Little effect on medial eyebrow, possibility of serious adverse events
Surgical methods					
Internal browpexy	Anchoring of the brow tissue (muscle and/or fat) to the periosteum of the frontal bone via a trans-blepharoplasty approach	To limit post blepharoplasty eyebrow descent	If formal brow "lifting" is expected	Avoids the cost and morbidity of more formal brow-lifting techniques	Modest efficasy, tenderness, and dimpling of the brow
Glabellar myoplasty	To transect the corrugator supercilii and procerus muscles during a blepharoplasty procedure	Complaints limited to glabellar folds and dermatochalasis	Limited forehead lift	Long-lasting improvement of vertical glabellar rhytids at the time of blepharoplasty	Supratrochlear neurovascular bundle is at risk
Direct brow lift	Elliptical incision immediately above the brows	Facial nerve palzy, men with recessed hairline, patients who can not undergo general anesthesia	If medial eyebow elevation is particularly sought	The greatest elevation per millimeter of excised tissue	A faint suprabrow scar
Tissue suspension with suture	Elevating the superficial soft tissue by self-anchoring sutures	Maybe suggested as a minimally invasive procedure	If patients asks for standard of care with proven long-term efficasy	Avoiding large incisions and greatly reducing recovery time	Little evidence on long-lasting aesthetic results
Coronal forehead and eyebrow lift	Coronal incision extends between the temporal fossae, behind the hairline, followed by extensive tissue excison/dissection and lift	Very heavy forehead with significant tissue excess and wrinkling	High hairline	Extensive incision with potentially persistent hair loss and numbness	High efficacy, no need for high-tech equipment
Endoscopic forehead Lift	Three to five small incisions withing the hair-bearing scalp, with titrated upper face dissection	Procedure of choice for patients with brow asymmetry	High hairline	Small incision with little risk of persistent hair loss and numbness	Longer learning curve, needs endoscope
Trichophytic forehead and brow lift	Superior incision marked along hairline and involves excision of bare forehead skin	Brow ptosis and high hairline	Short forehead	No need for general anesthesia, lowers the frontal hairline	Chance of scarring, brow asymmetry, and paresthesias of the forehead and scalp
Mid-forehead brow lift	Superior incision marked along a central forehead crease and then appropriate amount of tissue excised	Elderly men with significant brow ptosis that decreases superior visual field	Patients with unfurrowed forehead	No need for general anesthesia, lowers the frontal hairline	Prominent hyperemic scar, less effective for lateral brow ptosis

### Non-surgical methods

#### Botulinum toxin A (BTA) injection

**Figure 1:**
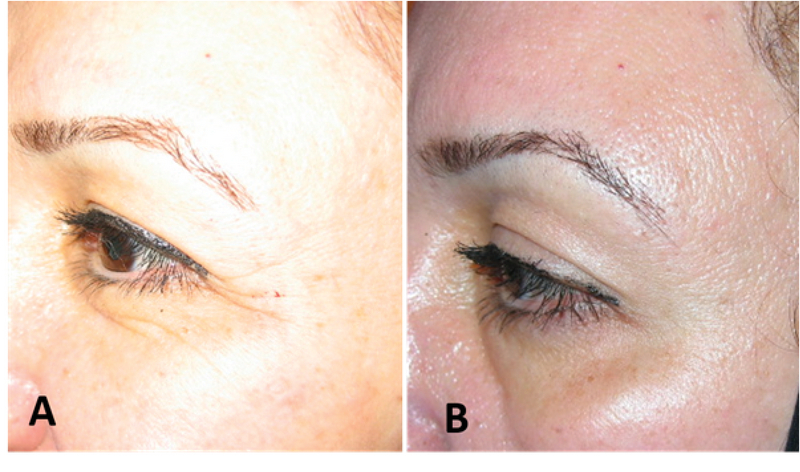
Before (A) and after (B) botulinum toxin A injection for the lateral eyebrow lift.

The brow depressors are the orbital orbicularis oculi (lateral and mid-brow), procerus (medial brow), corrugators (medial brow), and depressor supercilli (medial brow). BTA may be used for eyebrow lifting by targeting both glabellar and crow's feet areas.^[5]^ It mostly results in lateral eyebrow lift [Figure 1]. Ahn et al^[6]^ showed an average elevation in the central brow of 1 mm and of 4.8 mm in the lateral brow. The effect is temporary and lasts for three to six months.^[5]^ Pain, asymmetries, bruising and ecchymosis, erythema and edema, headache, diplopia, focal facial paralysis, dry eye syndrome, blepharoptosis, exaggeration of wrinkles, and muscle weakness are some of the adverse effects that might be seen by BTA injection for the upper face.^[5]^

### Soft tissue fillers

Fillers can enhance eyebrow contour and volume, and may be used for improving the elevation of the eyebrow tail in case BTA fails to provide desired eyebrow lifting.^[7]^ Hyaluronic Acid gels, the most frequently used fillers, are generally developed on the basis of two flow (rheological) parameters namely the G0 (elastic modulus, stiffness) and viscosity.^[8]^ High-G0 gels contribute to tissue vectoring (lifting) and resistance to deformation from the forces of gravity and facial movement. The viscosity of a gel is the ability to resist tissue spread. Thus, it contributes to contour stability. Fillers with high G0 and high viscosity lead to a stable lift and fill effect and are best implanted deep (pre-periosteal) for maximum effect.

**Figure 2:**
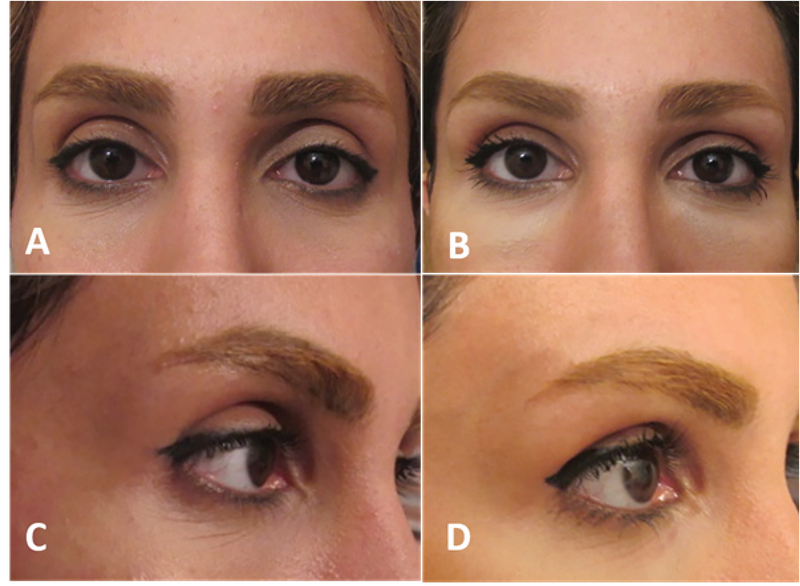
Before (A & C) and after (B & D) Hyaluronic acid gel (filler) injection under the lateral 2/3 of eyebrow. The injection yield to eyebrow projection especially in front view (B) and lift especially on lateral view (D).

To avoid inadvertent injection into the orbital cavity, one should first identify the orbital rim. The needle is positioned at the lateral end of the eyebrow; aspirated and then injected very slowly using a pre-periosteal bolus injection. Massage upward (to shape) is the next step. Injections in the lateral aspect of the eyebrows are intended to promote the support of the retro-orbicularis oculi fat [Figure 2]. Overcorrection of the eyebrow with filler can result in an unduly prominent eyebrow appearance or eyelid edema. The second injection should be given in the same manner medial to the first injection along the eyebrow. Supraorbital foramen should be respected.^[9]^

**Figure 3:**
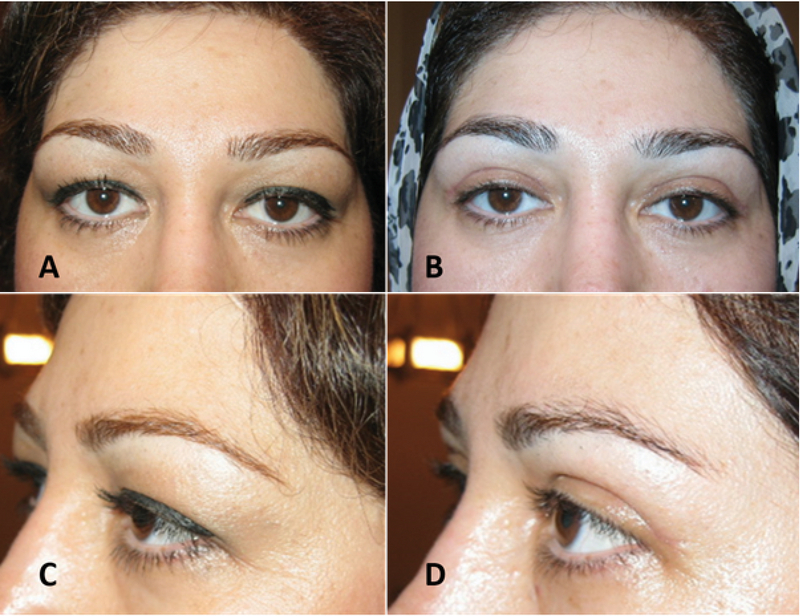
A gentleman before (A & C) and six months after (B & D) upper blepharoplasty and internal browpexy. Internal browpexy has resulted in no or minimal change in the eyebrow position.

Erythema and ecchymosis, foreign body granulomas, and migration of filler material are among the complications that may happen with fillers. Serious adverse events with soft tissue fillers include infection, biofilm reaction, cutaneous vascular compromise (skin/tissue necrosis), and most worrisome, blindness from retrograde injection into the ocular arterial system.^[10]^

### Surgical Methods

Surgical techniques [Table 1] generally either address the eyebrow ptosis alone (trans-blepharoplasty and direct approaches) or eyebrow and forehead together (trans-forehead approaches). In this section surgical techniques will be discussed in the following three categories: trans-blepharoplasty eyebrow lift, direct eyebrow lift, and trans-forehead eyebrow/forehead lift. While common complications of each procedure are listed, it should be noted that periorbital facial procedures may be followed by unexpected complications such as primary angle closure attacks.^[11]^ Thus realizing a few of most commonly encountered complications related to each procedure does not obviate the need to keep keen to thorough patient evaluation pre- and postoperatively.

### Trans-blepharoplasty approaches

#### Internal browpexy

Temporal brow ptosis is a common aging change that can contribute to upper eyelid fullness. Stabilizing or lifting the outer brow has become an essential adjunct to aesthetic upper blepharoplasty.^[12,13,14]^ A browpexy, or brow suture suspension, is not a formal lift. This technique provides a minimally invasive way to provide stabilization and possibly subtle elevation of the lateral brow.^[15]^ It consists in a measured anchoring of brow tissue (muscle and/or fat) to the periosteum of the frontal bone (or bone itself) above the superior orbital rim. The internal approach of the procedure was first described by McCord and Doxanas in 1990.^[14]^ In this original description, the sub-brow tissue is accessed through a blepharoplasty eyelid crease incision and the brow fat pad is dissected free of the frontal periosteum at a variable distance from the orbital rim. A guiding suture can be placed from skin to the internal wound to ensure the appropriate placement of the suspension suture on the undersurface of the brow soft tissue. The area of brow suspension to the frontal bone periosteum is measured directly. A 4-0 Prolene, for instance, engages the periosteum and also the internal brow tissue at the predetermined area (typically the inferior brow). Two to three similar sutures are placed laterally. When all sutures are tied, the brow is anchored to the new position [Figure 3].

Mokhtarzadeh et al reported that internal browpexy led to an average elevation in lateral/central brow position of 2.29 mm and 1.47 mm (average follow-up of 4–5 months).^[16]^ As such, this study among others,^[17,18]^ validate the ability of browpexy techniques to obtain a modest elevation (rather than mere stabilization) of eyebrow position. However, these studies generally lack long-term follow-up and hence do not present more than intermediate-term success. Future studies targeted at longevity of effect would be of great value.

Brassiere suture technique is a variant of internal brow approach, in which the orbicularis oculi is sutured to the superior lateral orbital rim periosteum.^[19,20]^ Such adjunct may enhance the projection and fullness of the eyebrow rather than its elevation.^[19]^ After standard blepharoplasty^[3]^ skin excision, the orbicularis muscle and orbital septum are fully opened from nasal to temporal. Two 5-0 chromic sutures are placed from the inferior wound edge orbicularis to the superior orbital rim periosteum and to the superior wound edge orbicularis.

On the other hand, some authors have advocated partial excision of ROOF to decrease eyebrow fullness which is called browplasty. After standard blepharoplasty and skin excision, the dissection is extended in submuscular plane toward the eyebrow. Once the dissection extends approximately 1–1.5 cm above the superior and lateral orbital rim, the brow fat pad should be clearly identified and an elliptical piece of brow fat pad should be removed approximately 1–1.5 cm in vertical dimension tapering nasally and temporally. The fat pad should be removed at the level of periosteum; however, periosteum should be left intact. This technique may cause temporary parenthesis of lateral two-third of the eyebrow.^[14]^ In our view, this technique mostly suits patients with heavy eyebrow especially male gender and patients with thyroid eye disease.

Internal browpexy may achieve less lift than desired and the sutures may cause dimpling of the overlying skin.^[21]^ Temporary forehead hypoesthesia (usually resolving in weeks to months) is a common adverse effect because superficial branches of the supratrochlear, supraorbital, and lacrimal nerves are often transected.^[18]^ Restricted brow movement and irregular contours are other causes of dissatisfaction.^[18]^

#### Glabellar myoplasty

**Figure 4:**
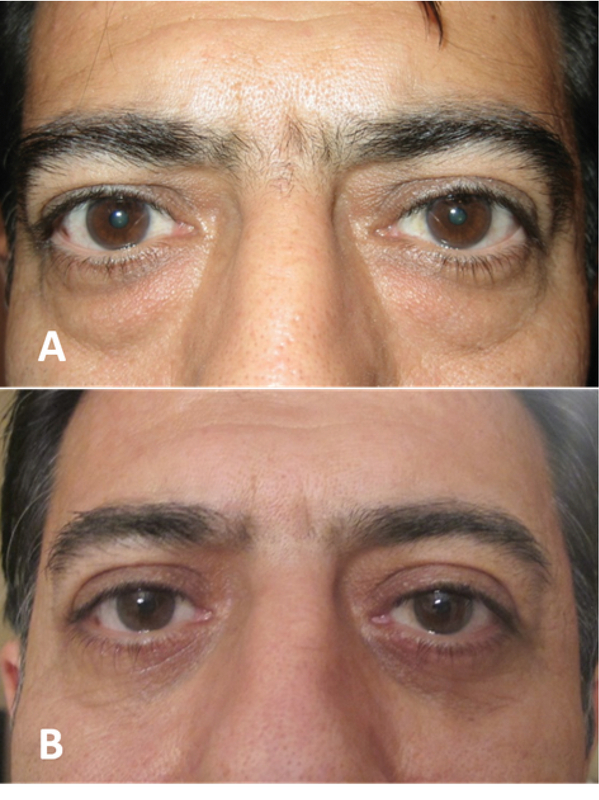
A patient before (A) and after (B) upper and lower blepharoplasty with trans-blepharoplasty corrugator excision. Trans-blepharoplasty glabellar myoplasty (corrugator muscle deactivation) leads to medial eyebrow lift.

A subset of male patients may be pleased with their brow position and shape, yet request long-lasting improvement of vertical glabellar rhytids at the time of blepharoplasty. An ideal approach in these patients may be blepharoplasty associated with glabellar myoplasty.^[22]^ Routine upper eyelid blepharoplasty is first performed and then attention is turned to the glabellar musculature. From the medial aspect of the upper eyelid compartment, dissection is then carried out superomedially in a supraperiosteal plane into the glabellar region. Care should be taken to avoid the supratrochlear neurovascular bundle. With retraction and direct visualization, the corrugator supercilii and procerus muscles are identified and transected with electrocautery. This procedure yields little if any improvement in brow position without a traditional brow lift procedure [Figure 4].

Glabellar myoplasty is usually ensued by more edema and ecchymosis compared to upper eyelid blepharoplasty, and less compared to conventional brow surgery.^[22]^ Occasional brow contour deformities may develop with overaggressive resection of muscle.^[22]^

### Direct Brow Lift

**Figure 5:**
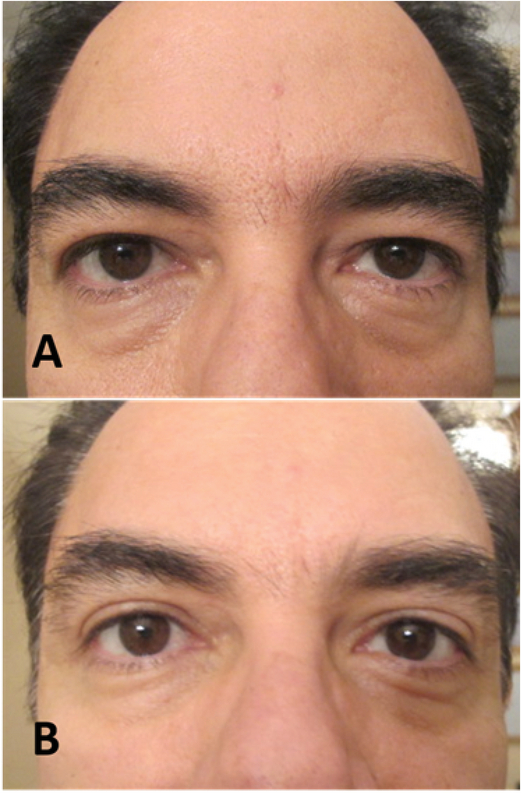
A subject with high hair line before (A) and after (B) direct eyebrow lift and upper blepharoplasty procedures. Direct eyebrow lift is mostly used for male with high frontal hair line which lateral eyebrow is lifted.

**Figure 6:**
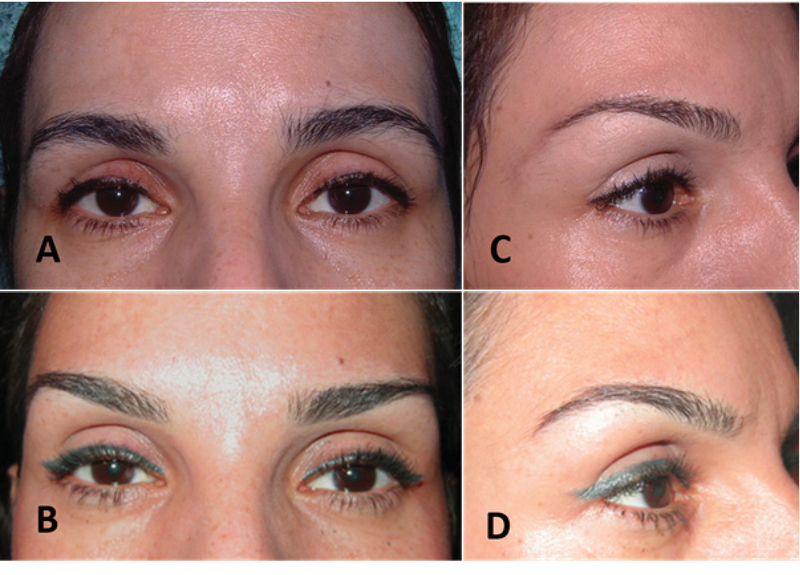
A very effective eyebrow tail lift with no visible scar (B & D) could be resulted from direct brow lift in female with high hairline (A & C).

The direct approach is most commonly used in patients with brow ptosis secondary to injury of the temporal branch of the seventh nerve.^[23]^ While cosmetic direct lift is mostly performed for men with recessed hairline who are thus not a good candidate for forehead-brow lift [Figure 5], it can be performed with good results in selected female patients who require only eyebrow tail elevation alone [Figure 6].^[24]^ This procedure is simple to perform and the amount of brow elevation is predictable. It maximizes the lift obtainable for a given amount of tissue removal. Direct brow lift involves bilateral elliptical incisions just above the brows. The inferior border of the incision is marked along the superior line of the eyebrow. After digital elevation to the desired height, the pen marks the site of desired elevation. The brow is released, and the forehead is marked at the level of the marker pen to designate the superior border of the ellipse.^[25,26]^ Dissection is carefully performed superficial to the frontalis muscle, avoiding damage to the supraorbital nerve and vessels. With the direct lift, descent of the brow postoperatively may limit efficacy, particularly with the use of absorbable sutures and if there is sever preoperative ptosis.^[21]^ Postoperative decent of the eyebrow mainly occurs within the first postoperative year.^[27]^ Scars that are depressed or hypertrophic may ensue at the superior edge of the brow in a small percentage of patients, potentially requiring revision or camouflage. Laser resurfacing will often improve the scar appearance and may also increase the elevation. Paresthesia and numbness associated with damage to the supraorbital nerve are common complications and typically resolve in a few months.^[21]^ Booth and colleagues reported that 74% (32/43 brows) of patients experienced numbness and that 7% were dissatisfied with this complication.^[25]^ Complications related to sutures include granuloma formation at the site of braided absorbable sutures and suture abscess formation.

**Figure 7:**
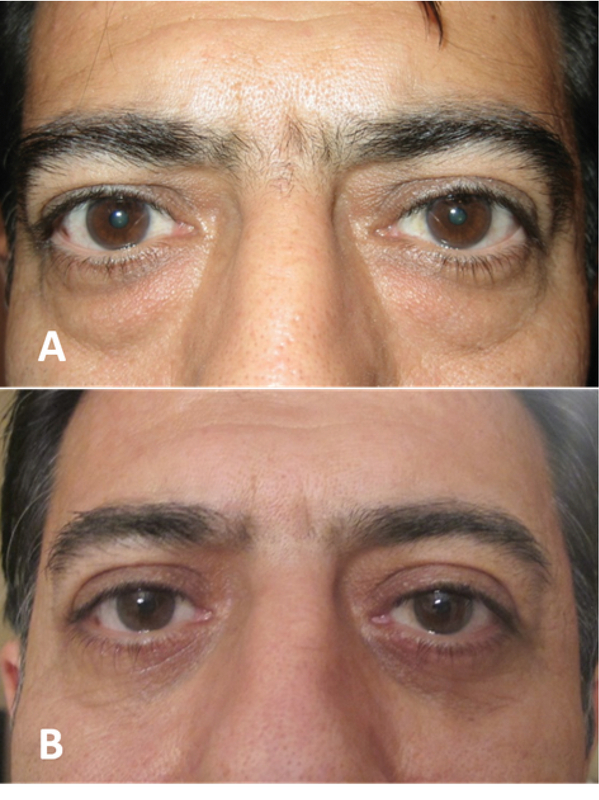
While suture (barbed) lift minimally elevates the eyebrow tail and consequently increased eyelid show (B & D), the effect is temporary. It is usually performed in young females (A & C) with realistic expectation.

### Trans-forehead Approaches

#### Suture lift

Sutures have been used to elevate lax skin of the neck and face, including the brow.^[28,29,30]^ With the advent of polypropylene barbed sutures, the capacity of sutures to withstand loads increased substantially.^[31]^ Facial lift by barbed sutures, with little or no soft tissue dissection, was first reported in the late 1980s by Russian authors Sulamanidze et al.^[32]^ These polypropylene self-anchoring sutures studded with numerous bidirectional (converging) barbs should be inserted into the subdermis, where the hook-like projections support and fix local tissue. Although numerous techniques evolved over the years, they all consist in the interposition of the soft tissues to the suture barbs, followed by inflammatory response and fibrosis [Figure 7]. The technique, in brief, involves a small incision made posterior to the hairline followed by undermining the forehead in the subcutaneous plane using a blunt-tipped cannula. Straight Keith needle with the attached thread is guided to exit just above the brow. The distal aspect of the sutures is trimmed so that the suture is beneath the skin. The Keith needle is removed, and the suture is locked into place. This is repeated along different spots, exiting above each eyebrow. The sutures are then contoured by stabilizing the threads and kneading the skin in a superior direction until there is significant elevation of the brow. There may be bunching of the skin at the hairline, but this resolves in three to six weeks. Proximally, the suture is tied down by taking a bite of fascia. The small incision is then closed.

Despite the continued use, evidence on long-term efficacy of thread lift is rare. Lycka et al followed-up 117 patients for 12–24 months and revealed (by blind photo evaluation) that 70% of the initial correction was maintained.^[28]^ Although early reports of thread lift were promising in terms of efficacy and safety (follow-up to 2.5 years),^[32,33]^ this was not consistently supported by ensuing articles. Some optimistic articles lacked a clear efficacy time frame^[34]^ or suffered from a short follow-up.^[35]^ In a recent systematic review,^[36]^ authors demonstrated that, within the past decade, little or no substantial evidence has been added to the peer-reviewed literature to support thread-lift sutures in terms of efficacy or safety. In fact, all included literature in the review, except two studies (prone to conflict of interest), demonstrated none or very limited durability of the lifting effect. In consequence long-term efficacy of thread lifting remains doubtful.^[37,38,39]^ Notable described complications of barbed suture include facial asymmetry,^[40]^ thread migration, and extrusion.^[41]^

#### Coronal forehead and eyebrow lift

**Figure 8:**
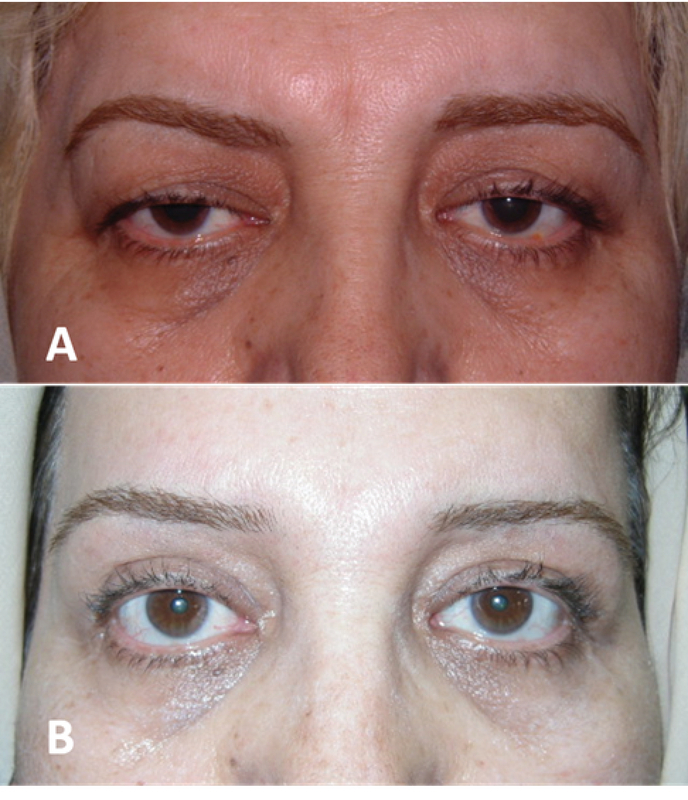
Before (A) and after (B) transcoronal lift with upper blepharoplasty and ptosis repair.

**Figure 9:**
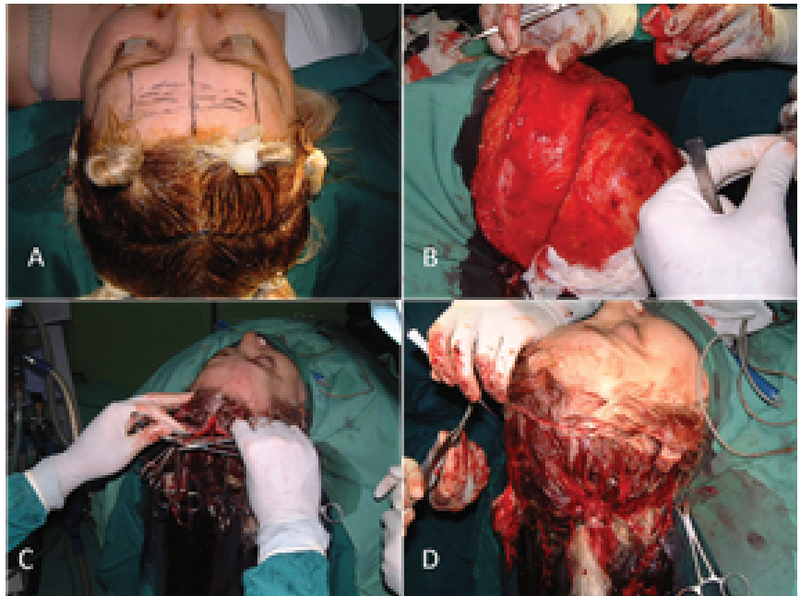
Cardinal steps of a transcoronal forehead lift include careful marking (A), complete release in the subperiosteal plane, up to the orbital rim (B), advancing the forehead flap over the scalp wound until the flap is moderately tight (C), and finally after excision of the excess segment and wound closure with staples (D).

The open coronal brow lift was the benchmark for eyebrow–forehead rejuvenation for several decades. It is highly effective in elevating and contouring the brows and removing the forehead–glabella rhytids [Figure 8]. It is however contraindicated in patients with high frontal hairline. The incision is usually made posterior to the hairline and extends between the temporal fossae [Figure 9(A)]. The dissection extends down to 2 cm above the supraorbital rim [Figure 9(B)].^[26]^ Most surgeons use a subgaleal dissection, 15% use subperiosteal dissection alone, and 12% use both planes of dissection.^[42]^ A procerus-corrugator myotomy is performed. is excised, and the wound is closed [Figure 9(C–D)]. Potential complications include skin necrosis, infection, hematoma, and noticeable scarring.^[43,44]^ The location of the incision perpendicular to the sensory nerves often compromises the sensory nerves, which results in temporary or potentially permanent numbness anterior and posterior to the incision.^[43,45]^ The theoretical complication of alopecia is often cited as a deterrent to the coronal open procedure; nevertheless, a survey found merely a small difference in the rates of alopecia between coronal and endoscopic approaches (4% vs 2.9%).^[46]^ Although other reports suggest that temporary alopecia may be as high as 33% in patients, typically resolves within three to six months.^[47]^

#### Endoscopic forehead lift

**Figure 10:**
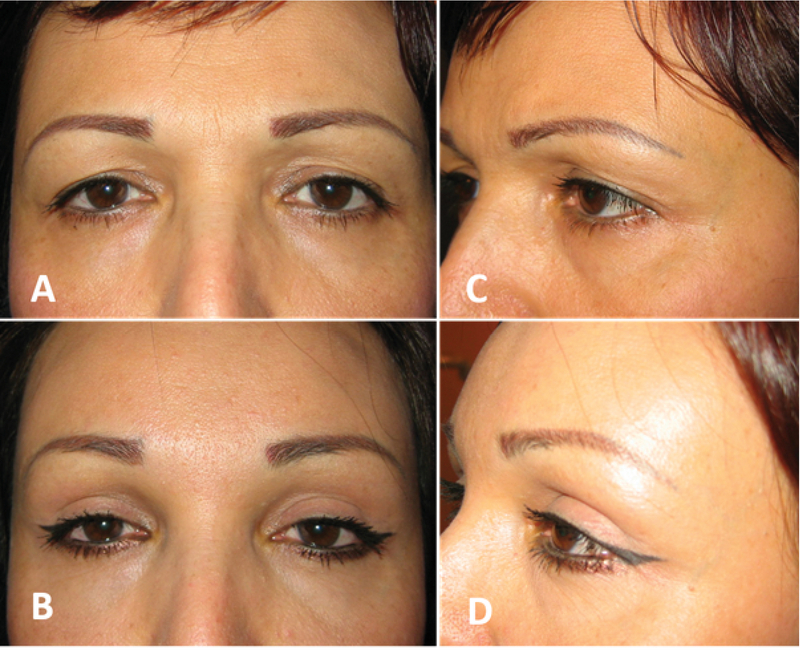
Before (A & C) and two years after (B & D) endoscopic forehead lift and upper blepharoplasty in a female in whom medial and lateral eyebrow lift are visible.

**Figure 11:**
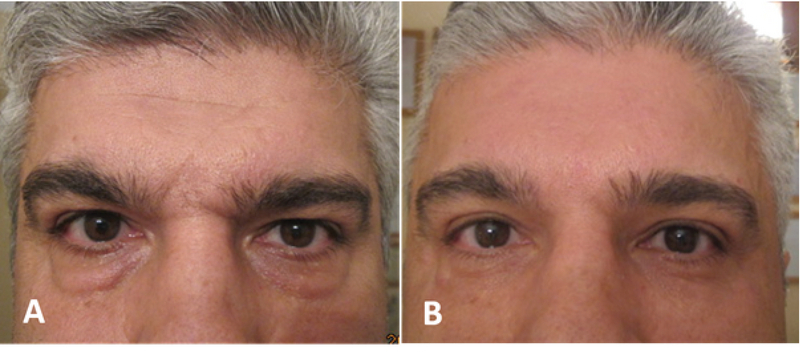
A male patient with good frontal hairline (A) who underwent endoscopic forehead lift and upper blepharoplasty procedures and ended up with a good eyebrow lift two and a half years after the surgery (B).

**Figure 12:**
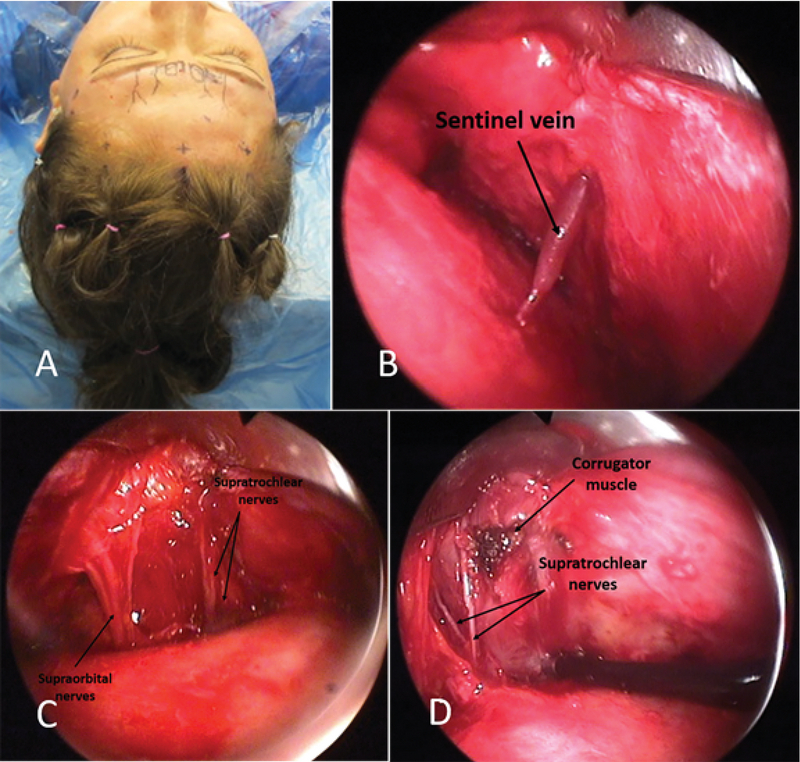
Endoscopic forehead lift. Novice surgeons are recommended to mark the location of corrugator muscles, supratrochlear and supraorbital neurovascular bundles before commencing the surgery (A). Sentinel vein is in close proximity to the frontal division of the facial nerve (B). Supratrochlear and supraorbital neurovascular bundles are readily identifiable during endoscopic lift (C). Corrugator muscle is disinserted and cauterized to smoothen the frown lines. Supratrochlear nerves crossing the muscle fibers should be preserved during the muscle disinsertion (D).

For most patients, an endoscopic brow lift is preferable to an open coronal brow lift because it is associated with reduced scarring and patient morbidity in women [Figure 10] and men [Figure 11].^[48,49]^ The endoscopic brow lift begins with marking the desired position of eyebrow elevation at the midline, lateral limbus, and lateral eyebrow. Five small scalp incisions, 1 to 2 cm in length, are typically made posterior to the hairline, three medially and two temporally.^[44]^ The medial incisions should be dissected dorsally, whereas the temporal incisions should be dissected toward the midline. In one survey, 39% of surgeons used a subperiosteal dissection, 13% used the subgaleal plane, and 12% used some combination of subperiosteal and subgaleal.^[46]^ Dissection may safely be performed without direct visualization up to 2 cm of the orbital rim. Thereafter endoscopic view allows for careful identification of the supraorbital vessels and nerves [Figure 12].^[50]^ While frontalis, corrugator, and procerus muscles are cut. Finally, the eyebrows are elevated, and tissue is fixed using a wide range of methods, including titanium or resorbable screws, fibrin glue, endotine, and suture with or without bone tunnels.

A survey of 21 plastic surgeons found that 70% of patients and 50% of plastic surgeons were satisfied with endoscopic brow lift results after at least two years of follow-up.^[51]^ Viksraitis and colleagues measured postoperative brow elevation using photographic and computer analysis.^[52]^ After at least six months of follow-up, median eyebrow elevation for 49 patients was 2.9 mm at the lateral canthus, 2.5 mm for the lateral limbus, 2.3 mm for the medial margin of the iris, and 2.2 mm at the medial canthus. Temporary injury may lead to temporary or persistent frontal numbness may follow.^[53]^ Postoperative headache was a common complaint in a study of 538 endoscopic brow lifts reported by Jones and colleagues.^[54]^

**Figure 13:**
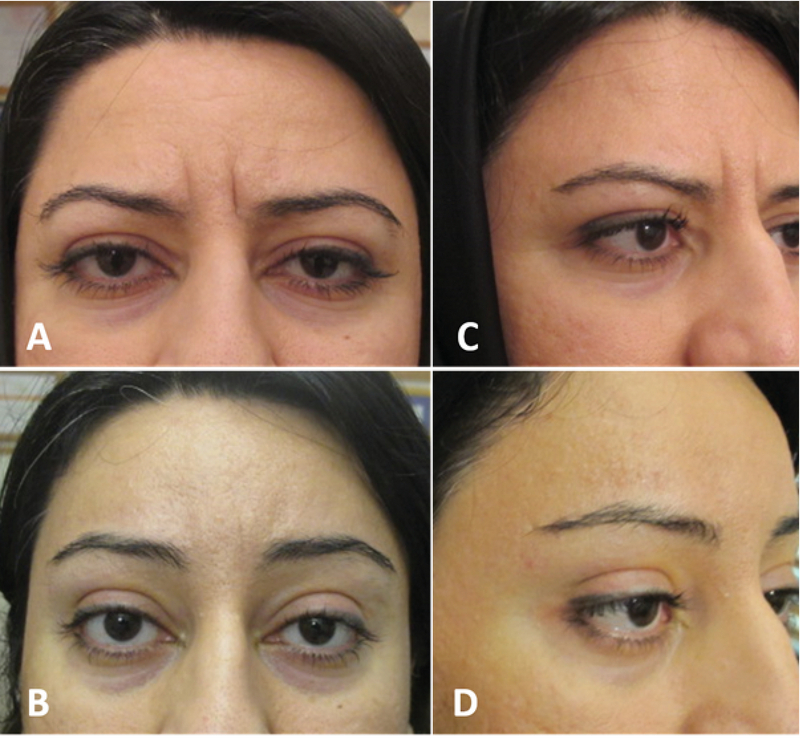
A patient with deep static glabellar lines (A & C) who underwent endoscopic forehead lift with glabellar smoothening and resulted in total eyebrow lift and no glabellar lines (B & D) one year after the procedure.

Endoscopic brow lift effectively elevates the medial, central, and lateral brow, and it seems to be the most effective technique for improving brow symmetry and may be the procedure of choice for patients with more than 1.5 mm difference in average brow height.^[55]^ Moreover Permanent smoothening of deep glabellar lines could be effectively achieved through endoscopic upper face lift [Figure 13].

A modification of coronal forehead lift has been described as minimally invasive, non-endoscopic brow lift. This technique applies the same dissection level (subperiosteal) and incisions (3 or 5 post hairline incisions) as the endoscopic approach but endoscop is not utilized. The anatomical location of the supratrochlear and supraorbital neurovascular bundles at the superior orbital rim should be identified and marked preoperatively (e.g. via palpation of the supraorbital notch). Tabatabai et al reported comparable results to endoscopic lift.^[56]^

#### Trichophytic forehead and brow lift

**Figure 14:**
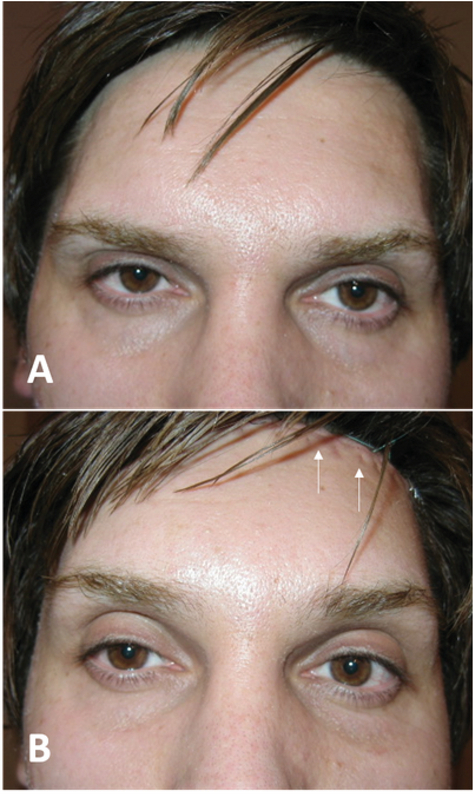
A young man with high hair line and total eyebrow ptosis (A) underwent pretrichial endoscopic forehead lift (B). While the lift effect is appreciated (B), scar of incision (arrows) will be visible for months and should be covered with hair styling.

In patients with a high hairline, forehead reduction as well as brow elevation may be achievable [Figure 14] via putting the incision at or 1-2 mm posterior to the hairline and excising the excess bare forehead later on in the procedure.^[57]^ To preserve the follicles at the edge of the wound (so that adequate hair is available for scar camouflage) the incision should be beveled in a posterior to anterior direction with minimal cauterization. The depth of incision and plane of dissection (subcutaneous, subgaleal, and subperiosteal) can be varied by surgeon's discren. The authors' preferred plane is the subperiosteal as it facilitates access to the corrugators and allows for better tissue release and hence maximal elevation. Dissection is continued anteriorly up to 1 cm above the superior orbital rim and along the lateral orbital rim down to the frontozygomatic suture line. Laterally, the dissection should proceed up to the conjoint tendon. Endoscope is helpful for safe dissection inferiorly below brow hairline^[58]^. Meticulous periosteal release and horizontal incision under direct visualization is crucial for an effective and symmetric elevation. The flap is then elevated to the desired level, and excess tissue is excised, followed by closure of the wound either by staples or sutures. The most common postoperative complications included scarring, brow asymmetry, and parenthesis of forehead and scalp.^[59]^

#### Mid-forehead brow lift

The mid-forehead brow lift is similar to the direct brow lift, except the incision is made in a deep transverse forehead crease rather than immediately above the eyebrow. This technique lowers the hairline by shortening the forehead. It is particularly effective in men with high or sparse frontal hairline and deep horizontal rhytids. The patient must be informed of the possible resultant visible scar for a long time. Dissection may be carried inferomedially to address the glabellar complex musculature, to improve central brow furrows. However, this procedure does not improve horizontal forehead creases.^[42]^

This review is an effort to provide an illustrated review of current popular techniques in brow lift. Although one may argue that limiting the search to PubMed or English language may have led to some personal techniques missed, the aim was to focus on the most commonly applied techniques (rather than trying to list all reported personal methods).

##  SUMMARY

The eyebrow complex is an integral aesthetic portion of the upper third of the face and the overall appearance of an individual. None of brow lift techniques are superior to the others. Therefore, facial surgeons should be familiar with all the techniques in order to recruit all in different subjects at different times. Non-surgical methods of forehead lift (BTA and filler) are temporary, need less experience, and in case of any complication, it is easier to correct. Trans-blepharoplasty and direct approaches can correct brow ptosis but not forehead wrinkles. A direct brow-lift is particularly useful in patients with facial palsy who need maximal elevation of the ptotic brow and are more likely to be accepting of the scar. Trans-forehead approaches can address both eyebrows and forehead. Currently, the most popular is the endoscopic approach, but some surgeons are not trained in this procedure, it requires special equipment and in some cases the final outcome is modest and/or not long-lasting.

##  Financial Support and Sponsorship

None.

##  Conflicts of Interest

There are no conflicts of interest.
